# Notes and News

**Published:** 1902-10-04

**Authors:** 


### Notes and News.

The late Mr. John Corbett has bequeathed £1,000 to the Worcester Infirmary.

A new out-patient block has been added to the South Devon and East Cornwall Hospital.

It is anticipated that structural alterations will have to be proceeded with at Netley Hospital, when the organisation has been remodelled, in order to bring it up to modern requirements.

The Hospital for Children with Hip Disease, long known as the Vine Hospital, is to be superseded by a new building to be opened on October 16th. The new hospital will contain 34 patients, and is arranged with central administration block and wards on either side. The site is most suitable.

The Manchester Skin Hospital is to be extended. The present buildings are to be enlarged, and new buildings for wards and offices erected. The work is estimated to cost between £30,000 and £35,000. The Finsen light is in use at the hospital now, but the demands for this treatment are far beyond the possibility of meeting them at the present time.

A new Workhouse Infirmary has been opened at Sunderland. It has been built much after the lines of the Dewsbury Workhouse Infirmary. The institution is divided into four separate buildings, one for the administration, one each for male and female patients, and one for maternity cases. The administration building contains an out-patients' department for the workhouse, a dispensary, and consulting-room. The general wards contain accommodation for 100 patients of each sex. The internal fittings, furnishing, and equipments are fully up to to a high modern standard. The total cost has been £40,000. The architects are Messrs. W. and T. R. Milburn.

A recent examination proved that 12 per cent. of the children of 36 public schools in New York city were suffering from contagious diseases of the eyes.

Mr. Stephen Paget, F.R.C.S., delivered the introductory address at the opening of the winter session at the Middlesex Hospital Medical School, on October 1st. Lord Sandhurst presented prizes to the students.

The foundation stone of the Smiley Cottage Hospital, at Larne, in Ireland, has been laid by Mrs. Smiley. Mr. Smiley has given £10,000 to establish the hospital. Mr. Fitzsimmons, the architect, is a local man. The accommodation will be for about 24 patients.

Hospital Saturday, in London, is to be held on October 11th this year. We hope the collection will show the steady increase of the past few years. The weekly collection in the workshops is not interfered with by the general collection from the public, and is continued until the end of the year.

The annual medical service at St. Paul's Cathedral, organised by the Guild of St. Luke, will take place this year on October 22nd, at 7.30 p.m., when the Bishop of Kensington will preach the sermon. A choir of over 200 voices, provided by the London Gregorian Choral Association, will render the music. Many members of the medical profession have intimated their intention of attending in academic robes, so the service will be of its usual brilliant character. Admission to the space under the dome will be by tickets only.

The loss and inconvenience which may be caused to a community by even a comparatively slight outbreak of small-pox are well illustrated by recent events in Barbados. That island is suffering from an outbreak of small-pox of a mild type. Yet the disease has already proved a serious incubus to the trade of the place owing to the strict quarantine which has been imposed against it by the neighbouring colonies, all the islands except Trinidad and the colony of British Guiana having prohibited the landing of any goods whatever coming from Barbados. The steamers have ceased to communicate directly with the shore, and boatmen, lightermen, and wharfmen are out of employment. Yet small-pox is the very type of a preventable disease.

Among the forms of municipal socialism which we imagine even the *Times* would allow to be permissible is that which is involved in a health resort advertising its own virtues. Such a town lives by its visitors, who are indeed the breath of life to the whole community, and a corporation may well spend a trifle out of the rates in letting the outer world know all about its advantages. Even the most ardent economist we should imagine would not object to the municipal enterprise which has led to the publication of the very pretty little pamphlet entitled "Buxton: its History, Waters, Climate, Scenery, etc.," a copy of which has recently been sent to us, a pamphlet which shows that Buxton is attractive to many beside the rheumatic folk who swear so confidently by the virtues of its baths.

